# Cloning, computational analysis and expression profiling of steroid 5 alpha-reductase 1 (SRD5A1) gene during reproductive phases and ovatide stimulation in endangered catfish, *Clarias magur*

**DOI:** 10.1038/s41598-023-46969-1

**Published:** 2023-11-09

**Authors:** Deepak Agarwal, Gulshan Kumar, Mohd Ashraf Rather, Ishtiyaq Ahmad

**Affiliations:** 1 Institute of Fisheries Post Graduate Studies, TNJFU, Kazhipattur, India; 2 College of Fisheries, Gumla, Phasia, India; 3 Division of Fish Genetics and Biotechnology, Faculty of Fisheries, SKUAST-Kashmir, Srinagar, India

**Keywords:** Biotechnology, Structural biology

## Abstract

The cloning and characterization of the complete coding sequence of the *Clarias magur* SRD5A1 (CmSRD5A1) gene, which encodes an enzyme responsible for regulating steroid levels by converting testosterone into 5α-dihydrotestosterone (DHT), have been successfully achieved. DHT plays a vital role in enabling the complete expression of testosterone's actions in neuroendocrine tissues. The ORF of the full-length cDNA sequence of SRD5A1 was 795 bp, translating into 265 amino acids, with a total length of 836 bp including UTRs. Like other vertebrates, the signal peptide analysis revealed that SRD5A1 is a non-secretory protein, and hydropathy profiles indicated that it is hydrophobic in nature. The 3D structure of CmSRD5A1 sequence generated above was predicted using highly accurate AlphaFold 2 in Google Colab online platform. CmSRD5A1 contains seven transmembrane helices connected by six loops, with the N-termini located on the periplasmic side and C-termini on the cytosolic side. Structural superimposition with known bacterial and human SRD5As showed very high structural similarity. The electrostatic potential calculation and surface analysis of CmSRD5A1 revealed the presence of a large cavity with two openings one highly electropositive towards the cytosolic side and another relatively neutral towards the transmembrane region. The structural comparison revealed that the electropositive side of the cavity should bind to NADPH and the steroid hormone in the hydrophobic environment. Polar residues binding to NADPH are highly conserved and the same as known strictures. The conserved residues involved in hydrogen bonding with the ketone group at C-3 in the steroids hence fevering Δ4 double-bond reduction are identified as E66 and Y101. Our findings showed that SRD5A1 expression was lower during the spawning phase than the preparatory phase in female fish, while the administration of Ovatide (a GnRH analogue) resulted in up-regulation of expression after 6 h of injection in the ovary. In males, the lowest expression was observed during the preparatory phase and peaked at 16 h post- Ovatide injection in the testis. The expression of SRD5A1 in the brain of female fish was slightly higher during the Ovatide stimulation phase than the spawning phase. This study represents the first report on the cloning and characterization of the full-length cDNA of SRD5A1 in Indian catfish.

## Introduction

In neuroendocrine tissues, the metabolism of testosterone (T) involves two biologically significant pathways regulated by brain aromatase and 5α-Reductase. Aromatase, which is a critical enzymatic complex, plays a key role in the last step of the synthetic steroidogenic pathway by converting aromatizable androgens into estrogens. In several fish species, two aromatase genes have been identified: the cyp19a1a gene that codes for aromatase A, primarily expressed in the gonads, and the cyp19a1b gene that codes for aromatase B, mainly expressed in the brain^1–5^. On the other hand, 5α-reductase, also known as 3-oxo-5-α-steroid 4-dehydrogenases, is a microsomal enzyme that reduces the C4–C5 double bond of various steroid substrates^6,7^. It converts testosterone into the more potent androgen 5α-dihydrotestosterone (DHT)^8^. In rodents, monkeys, humans, and fishes (including zebrafish and medaka), two isozymes of 5α-reductase encoded by distinct genes, namely 5αR1 and 5αR2, have been identified^9–11^. The PMET (Phospholipid methyltransferase) superfamily encompasses 5α-reductase, a microsomal enzyme responsible for the conversion of testosterone into dihydrotestosterone which is a crucial reaction for androgen action, contributing to the formation of the male phenotype during embryogenesis and the growth of tissues like the prostate in humans^12^. Although aromatase in fish brain has been widely studied, limited reports exist for 5α-reductase^13,14^. The steroidogenic metabolite DHT, produced by 5α-reductase, has received little attention in non-mammalian species, as it is thought to be relatively biologically inactive in fishes. However, recent studies have demonstrated the potency of DHT as an androgen in fish^15^. Moreover, the 5α-reductase has been suggested to be necessary for the full expression of testosterone actions in neuroendocrine tissues^16^. Despite its importance, the 5α-reductase gene has not yet been characterized in fishes of the Clariidae family, and no earlier studies are available in any species of walking catfish.

*Clarias magur*, commonly known as the Indian walking catfish, is a seasonal breeder that reproduces during the rainy season from July to August^17^. In captivity, obtaining milt from male silurid species, including *Clarias magur*, is difficult and requires sacrificing the brooders^18^. In contrast, this fish breeds easily in its natural environment. The underlying physiological mechanisms of reproduction in this species remain largely unexplored. Despite a few reports on genes involved in reproductive physiology^19–25^, there is a lack of information on the molecular mechanisms involved in spawning. Therefore, the present study aims to characterize the coding sequences of 5α-reductase in *Clarias magur* and investigate its expression profiling at different reproductive stages to explore its potential role in spawning. This investigation will provide new insights into the reproductive biology of *Clarias magur* and could contribute to the development of improved breeding strategies for this economically important fish species.

## Materials and methods

### Sample and tissue collection

The wet lab experiment was conducted at the Freshwater Fish Farm, ICAR-CIFE, Powarkeda, Madhya Pradesh, India. Four reproductive stages of *C. magur* were examined: (i) preparatory, (ii) mature, (iii) 6 h post Ovatide injection, and (iv) 16 h post Ovatide injection. A total of six male and six female live fish were sampled for each stage, with sampling performed based on the maturation month of the fish. For example, fish in the preparatory stage were sampled in February, while those in the mature stage were sampled in August^17^. All experimental protocols were approved by ICAR- Central Institute of Fisheries Education, Mumbai. To aesthesis the fish, clove oil @ 0.05 mL per 500 mL of water was used. To examine the reproductive genes profile, Ovatide hormone (Hemmo Pharmaceuticals Pvt. Ltd., Mumbai) was administered to male and female fishes at a rate of 0.5 ml kg^−1^ and 1.0 ml kg^−1^ per body weight^26^, respectively. Sampling was carried out after 6 h and 16 h, and both brain and gonadal tissues were collected in RNAlater (QIAGEN, USA) and stored at − 80 °C until further analysis. RNA was extracted from tissue pools comprising three specimens.

### Total RNA extraction and cDNA conversion

The total RNA from the tissue samples was extracted using Trizol reagent (Invitrogen, USA) following the manufacturer's guidelines. Genomic DNA contamination was removed from the extracted RNA (5 µg) by treating it with DNase I (Thermo Scientific, USA). The purified RNA was evaluated for quality and quantity using gel electrophoresis on 1% Agarose and NanoDrop spectrophotometer (Thermo Scientific, USA). To synthesize cDNA, DNase I-treated total RNA (1 µg) was reverse transcribed using Oligo dT primer and RevertAid reverse transcriptase First Strand cDNA Synthesis kit (Thermo Scientific, USA) according to the manufacturer's instructions^19^.

### Molecular cloning and sequence analysis of SRD5A1

The conserved regions of SRD5A1 genes were identified by multiple sequence alignment with orthologous sequences, and primers were designed against them through Primer3 plus online primer designing tool (Table 1) and synthesised from Eurofins Genomics India Pvt. Ltd. For amplification, the PCR was performed in a 96-well Takara PCR System with a total volume of 25 µL. The obtained partial cDNA fragment of 382 bp was eluted from the gel, cloned into pTZ57R/T vector, sequenced and confirmed by BLASTn similarity search. For RACE PCR, gene-specific primers (Table 1) were designed from the identified partial sequence. The reaction was carried out for both 3′ and 5′ ends to obtain the full-length cDNA sequence of the gene comprising of the open reading frame (ORF), 3′ and 5′ untranslated (UTR) regions. The desired PCR product was purified, cloned, and sequenced, and the sequence was confirmed by BLASTn software available at http://www.ncbi.nlm.nih.gov/blast. The annealing temperature of each primer set was optimized by gradient PCR. The reaction mixture contained 2.5 µL of 10 × Taq buffer, 0.5 µL of 10 mM of each dNTP mix, 2.5 µL of 25 mM MgCl2, 1 µL each of sense and antisense primers (10 pmol), 1 µL of cDNA (500 ng), 0.25 µL of 5 U/µL Taq DNA polymerase, and 16.25 µL of nuclease-free water^19^.

**Table 1:** Primers used in the study.

Primer name	Sequence (5ʹ–3ʹ)	Usage
CmSRD5A1F	TCGAGGAGGAAAGCCCACAC	Partial cDNA amplification
CmSRD5A1R	GGCTCGACTGGAAAGGACGA	Partial cDNA amplification
GSP1 3ʹ	CTTCCCTAAGGGTTGGGTTTCACATCC	3ʹ RACE for CmSRD5A1
GSP2 3ʹ	CACAGTGTTGCCTTTGCTTTCTTCACC	3ʹ RACE for CmSRD5A1
GSP1 5ʹ	CTCAAAGGCAGAAATAACGCAGGCAGC	5ʹ RACE for CmSRD5A1
GSP2 5ʹ	GCCAGAGCCATGAGAGTCATTAAATAC	5ʹ RACE for CmSRD5A1
CmSRD5A1F_qRT	TGGAGGACATTCTGTTGGGGCTGT	Real time PCR
CmSRD5A1R_qRT	CACACGCTGCCAGAGCCATGAG	Real time PCR
B_actinF	GCCGAGAGGGAAATTGTCCGTG	Internal control
B_actinR	GCCAATGGTGATGACCTGTCCG	Internal control

### ORF identification and Amino acid sequence prediction

The open reading frame (ORF) of the obtained full-length cDNA sequence was identified using the NCBI ORFfinder tool. Gene Runner software was used to deduce the amino acid sequence of the coding region and conserved domains in the deduced amino acid sequence were identified using the NCBI Conserved Domain Database (CDD) search tool (https://www.ncbi.nlm.nih.gov/Structure/cdd/wrpsb.cgi)^19^.

### Evolutionary analysis

The amino acid sequence of SRD5A1 from various species including *C. magur* (Indian magur), *I. punctatus* (Channel catfish), *L. calcarifer* (Sea bass), *P. nattereri* (Piranha), *D. rerio* (Zebrafish), *C. carpio* (Common carp), *S. salar* (Atlantic salmon), *Homo sapiens* (human), *Mus musculus* (Mouse) and *Bos taurus* (Cattle) was analyzed for multiple sequence alignment using the CLC Genomics Workbench software (QIAGEN, USA). The resulting alignment was utilized to construct a Neighbor-joining tree using MEGA 11.0 software^27^ with 1000 bootstrap replicates.

### In silico analysis and 3D structure prediction of CmSRD5A1

The deduced amino acid sequence of CmSRD5A1 underwent analysis using various tools. Physiochemical parameters were evaluated using the ProtParam tool at ExPASy^28^. The secondary structure was predicted through online tools including PSIPRED V.4.0 (http://bioinf.cs.ucl.ac.uk/psipred/) and SOPMA^29^. The protein's hydropathy profile was examined using the ProtScale program^28^. Furthermore, signal peptides were predicted utilizing SignalP 4.0 software^30^. The AlphaFold 2 software^31^ is available on the ColabFold platform (https://colab.research.google.com) and was utilized to predict the 3D structure of the CmSRD5A1 deduced sequence (accession no. BBE00816.1). AlphaFold is a highly accurate neural network-based tool for protein structure prediction, employing training procedures that incorporate evolutionary, physical, and geometric constraints of protein structures. In the 14th Critical Assessment of Protein Structure Prediction (CASP14) competition, AlphaFold demonstrated accuracy comparable to experimental structures in most cases and significantly outperformed other prediction methods^32^. ColabFold, hosted on Google's Jupyter Notebook platform^33^, provides a free and accessible environment for performing protein structure prediction by combining the fast homology search method MMseqs2 with AlphaFold2 or RoseTTAFold, thereby accelerating the prediction process^34^. The structure was predicted in no template mode. The top-ranked structure was relaxed using amber. Multiple sequence alignment (MSA) options were kept as msa_mode: mmseqs2_uniref_env and pair_mode: unpaired_paired (default settings). Parameters of advanced and sample settings were kept as auto. Post-translational modifications (PTM) were not considered during structural prediction as no PTMs were reported for this enzyme in available literatures.

### Model evaluation and Protein–protein interactions of CmSRD5A1

The quality assessment of the predicted 3D structure of CmSRD5A1 involved the analysis of the Ramachandran plot and AlphaFold quality estimates. The Ramachandran plot illustrates the structure's geometry by representing torsion angles on a residue-wise basis. To evaluate the quality, the Procheck tool^35^ available on SAVESv6.0 (https://saves.mbi.ucla.edu/) was utilized. This tool provides information on the total number and percentage of residues distributed in allowed and disallowed regions of the plot.

AlphaFold, on the other hand, assesses errors in predicted structures using two approaches: one that verifies if a residue is correctly positioned within its local environment, and another that determines if domains are accurately positioned relative to each other^36^. The predicted local distance difference test (pLDDT) score, ranging from 0 to 100, serves as a per-residue confidence score. A pLDDT score above 90 indicates a high level of confidence, while a score below 50 suggests low confidence. This score estimates whether the predicted residue's distances to neighbouring C-alpha atoms (within 15 Angstroms) align with the distances observed in the true structure. The study also found that a pLDDT < 50 is a reliable indicator of disorder, implying that such a region is likely to be unstructured in physiological conditions or only structured when part of a complex^36^. Another metric used in the study is the Predicted Aligned Error (PAE), which measures the distance error between pairs of residues^36^. PAE provides AlphaFold's estimation of the positional error at residue x when aligning the predicted and true structures based on residue y. These values typically range from 0 to 35 Å and are represented as a heatmap image, where the vertical and horizontal axes correspond to residue numbers and the color at each pixel indicates the PAE value for the corresponding pair of residues. When the relative position of two domains is confidently predicted, the PAE values for residue pairs involving one residue from each domain tend to be low (less than 5 Å).

**Figure 1:**
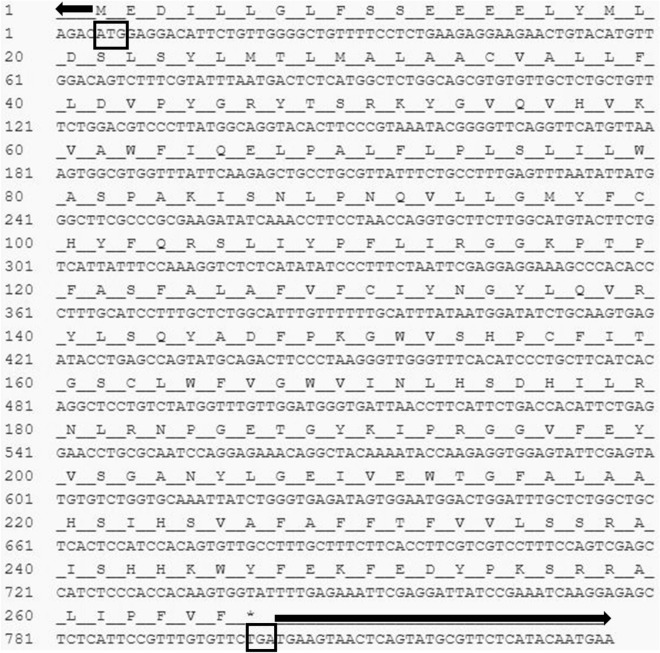
Nucleotide and the deduced amino acid sequence of full-length cDNA of *Cm*SRD5A1. The total number of nucleotides 836 bp. Arrows indicate 5ʹ UTR and 3ʹ UTR and boxes indicate the start and stop codons.

The protein–protein interactions of the *Cm*SRD5A1 protein with other proteins involved in the steroidogenesis pathway were determined using the online software STRING^37^, which predicted the interactions (Fig. 1).

### Structural comparison, ligand interaction and visualization

The enzymes 3D structures were analyzed using UCSF-Chimera software^38^. To superimpose CmSRD5A1 onto human SRD5A2 (HsSRD5A2; PDB ID: 7bw1) and SRD5A of Proteobacteria bacterium (PbSRD5; PDB ID: 7c83), the Matchmaker tool of Chimera software was utilized, with the latter serving as the reference structure. The alignment algorithm employed was Needleman-Wunsch with the BLOSUM-62 matrix. Atom pairs exceeding 2.0 Å were pruned iteratively until none remained. Following the superimposition, a structure-based sequence alignment was generated, setting a residue-residue cut-off of 5 Å. NADPH and NADP-dihydrofinasteride adduct molecules were then added to the CmSRD5A1 structure. Ligand binding and catalytic residues were identified in the CmSRD5A1 structure based on the structure-based sequence alignment. Additionally, various images depicting the structure and ligand interactions were generated.

### Calculation of electrostatic potential

The 3D structure of CmSRD5A1 was initially prepared using the PDB2PQR tool available on the APBS-PDB2PQR server (https://server.poissonboltzmann.org/). The resulting PDB2PQR output was then utilized to calculate the electrostatic potential through the APBSA tool in auto-mode. APBS (Adaptive Poisson-Boltzmann Solver) is an electrostatics solver that addresses the equations of continuum electrostatics for large biomolecular assemblies. The electrostatic potential obtained from the PDB2PQR server was visualized using the server's provided tool. To generate colored images of the CmSRD5A1 surface, UCSF-Chimera was employed with three color combinations: − 10 kcal/(mol^−e^) represented in red, 0 kcal/(mol^−e^) in white, and + 10 kcal/(mol^−e^) in blue.

### CmSRD5A1 mRNA expression analysis

The basal mRNA expression analysis of SRD5A1 in the selected tissues of *C. magur* was conducted using the LightCycler 450 Real-time PCR detection system (Roche, USA). Each reaction consisted of a 10 µL reaction mix, which included 5 µL of 2 × Maxima SYBR Green qPCR master mix (Thermo Scientific, USA), 0.5 µL of each gene-specific primer (0.3 pM) (Table 1), 2 µL of cDNA (20 ng), and 2 µL of nuclease-free water. The PCR amplification utilized the default thermal profile, commencing with an initial denaturation step at 95 °C for 10 min, followed by 40 cycles of denaturation at 95 °C for 20 s, annealing at 65 °C for 20 s, and extension at 72 °C for 30 s. To ensure accuracy, all reactions were run in triplicate and the experiment was repeated twice. The relative expression of the target gene was determined using the comparative Ct method (2^−ΔΔCt^)^39^ with β-actin as the housekeeping gene for gene normalization^19^.

### Ethics statement

The guidelines of the CPCSEA (Committee for the Purpose of Control and Supervision of Experiments on Animals), Ministry of Environment and Forests (Animal Welfare Division), Government of India on care and use of animals in scientific research were followed to care and rear the animals used in the present study. The study was approved by the Board of studies and authorities of ICAR-Central Institute of Fisheries Education (ICAR- CIFE), Mumbai-61.

### Statistical analysis

The one-way analysis of variance (ANOVA) of statistical package SPSS 17.0 (USA) was used to test the statistical significance of differences in mRNA transcript levels. P < 0.05 was considered statistically significant. The results were expressed as mean ± standard error (bars).

### ARRIVE guidelines

The present study has been carried out in accordance with ARRIVE guidelines.

## Results

### Molecular cloning of CmSRD5A1 gene

**Figure 2:**
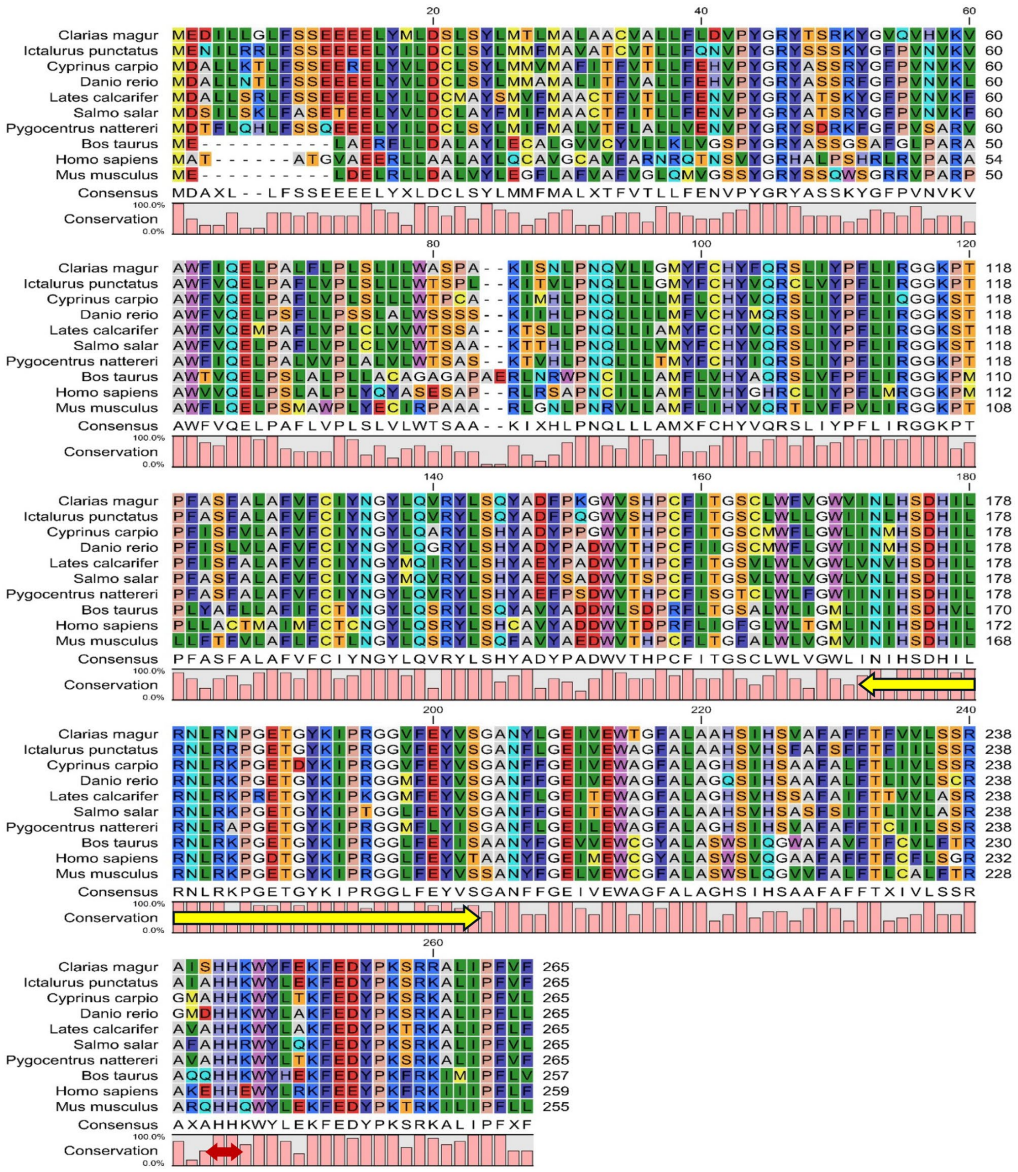
Conserved amino acid sequences of the SRD5A1 protein across different vertebrates. Amino acid sequences encoding the SRD5A1 protein were aligned using the Clustal W algorithm of the CLC Genomics Workbench. The bars below indicate the conserved motifs in different vertebrates. Brown arrow represents the histidine residues. Yellow arrow represents the highly conserved NADPH-binding domain. The amino acid sequences of *I. punctatus* (XP_017336166.1), *C. carpio* (XP_042632703.1), *D. rerio* (XP_009292518.1), *L. calcarifer* (XP_018556926.1), *S. salar* (XP_014033435.1), *P. nattereri* (XP_017566885.1), *B. Taurus* (NP_001092607.1), *H. sepiens* (NP_001038.1) and *M. musculus* (NP_780492.2) were retrieved from NCBI database.

**Figure 3:**
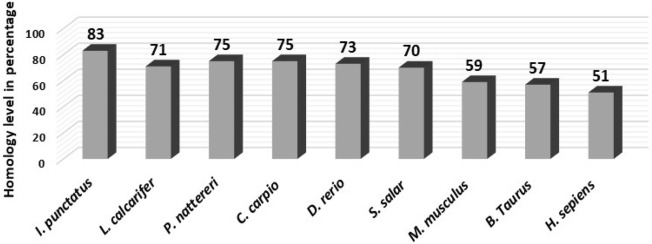
Homology level of amino acid sequences of different species.

**Figure 4:**
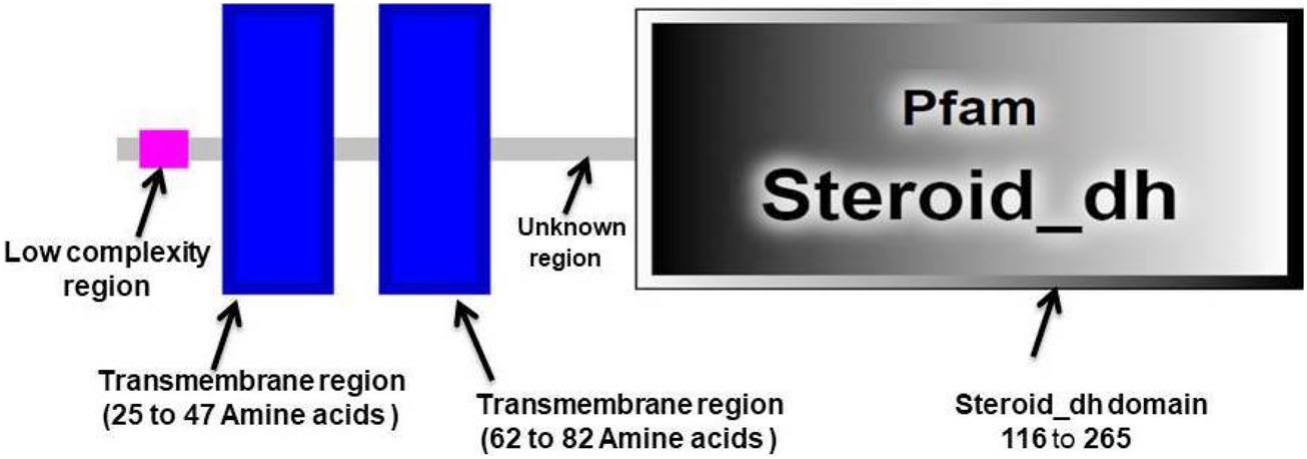
Conserved domains of CmSRD5A1 protein.

A 382 bp partial cDNA fragment of CmSRD5A1 was successfully cloned from the brain of *C. magur*. Employing the 5'/3' RACE-PCR technique, a full-length cDNA fragment of 836 bp including UTRs (ORF 795 bp) was obtained for CmSRD5A1 (Fig. 2), which encoded a putative protein consisting of 265 amino acids. Figure 3 presents the ORF of the CmSRD5A1 protein, along with its signature domains. The complete CDS sequence derived from this study has been submitted to NCBI GenBank (Accession number: LC383722 {Protein Id: BBE00816}). Comparative analysis of the amino acid sequence of CmSRD5A1 obtained in this study with similar proteins documented in NCBI indicated a substantial level of similarity (Fig. 4). The amino acid sequence exhibited the highest similarity with *Ictalurus punctatus* (83%), followed by *Pygocentrus nattereri* and *Cyprinus carpio* (75%).

### In silico analysis of CmSRD5A1 amino acid sequences

**Figure 5:**
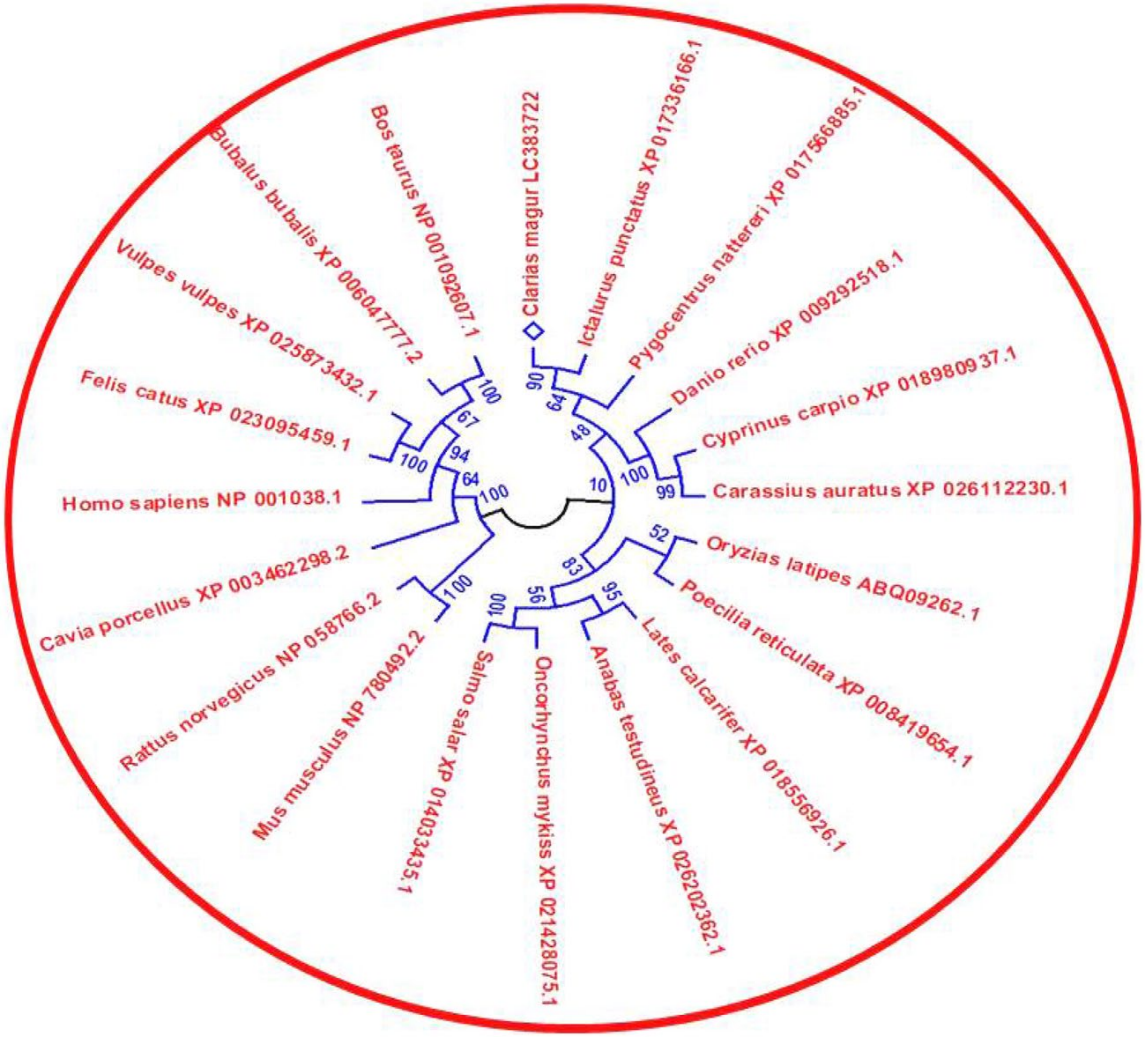
Phylogenetic analysis of CmSRD5A1.

**Figure 6:**
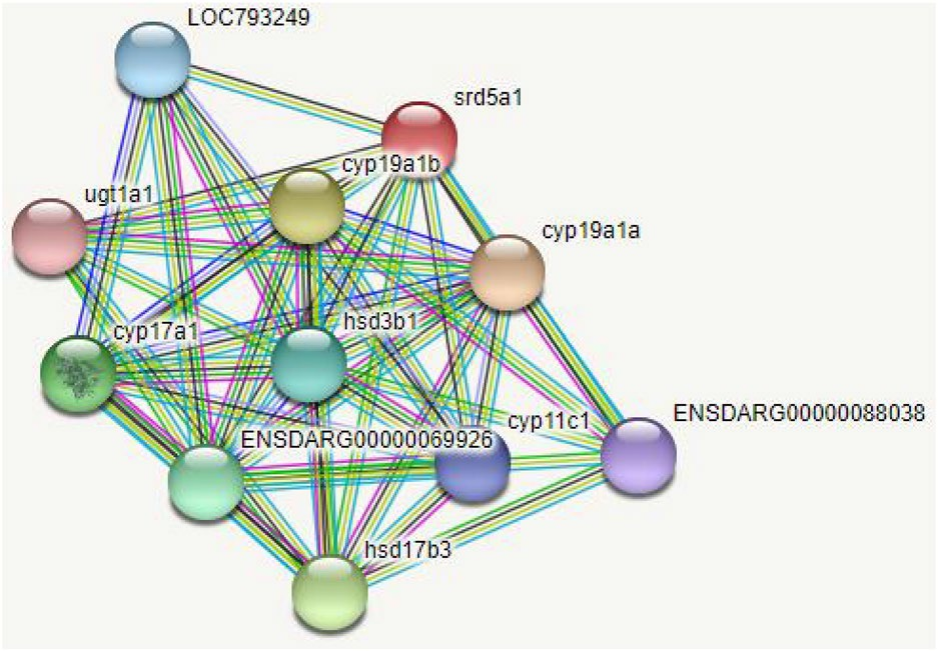
Protein–Protein interaction of CmSRD5A1 with different proteins.

The multiple sequence alignment of the deduced amino acid sequence of CmSRD5A1 with sequences from other species revealed well-conserved regions across the sequences (Fig. 3). Furthermore, the CDD search analysis identified two transmembrane regions and a highly conserved steroid_dh domain spanning amino acids 116–265 in the CmSRD5A1 protein, exhibiting a remarkable E-value of 2.06e^−63^ across all species included in the multiple alignment analysis (Fig. 5). The Neighbour-joining tree constructed based on the SRD5A1 protein distinguished closely related species into separate clades. The catfish species formed one distinct cluster, while the cyprinids formed another separate group (Fig. 6). Bovine species clustered together, representing a distinct group, while mammals and humans formed a sister group. Notably, the CmSRD5A1 protein displayed close relatedness to the channel catfish and Piranha, followed by the cyprinids. This finding aligns with the classification and evolutionary status of the species.

### Secondary structure analysis of CmSRD5A1 protein

The CmSRD5A1 protein was shown to have an apparent molecular weight (MW) of 30.428 Kilodaltons with a pI of 8.21. The secondary structure of CmSRD5A1 predicted by the PSIPRED server showed 10 α-helices, 11 coils and no β-sheets in the protein. No signal peptide could be identified in CmSRD5A1 by SignalP 4.1 server. The hydropathy analysis of the CmSRD5A1 peptide chains revealed the hydrophobic nature of the protein. CmSRD5A1 protein exhibited 16 serine (Ser), 3 threonines (Thr), and 5 tyrosine (Tyr) phosphorylation sites that are uniformly distributed throughout the polypeptide chain as predicted by NetPhos 3.1 Server.

### 3D structure prediction, superimposition, quality evaluation and electrostatic potential

**Figure 7:**
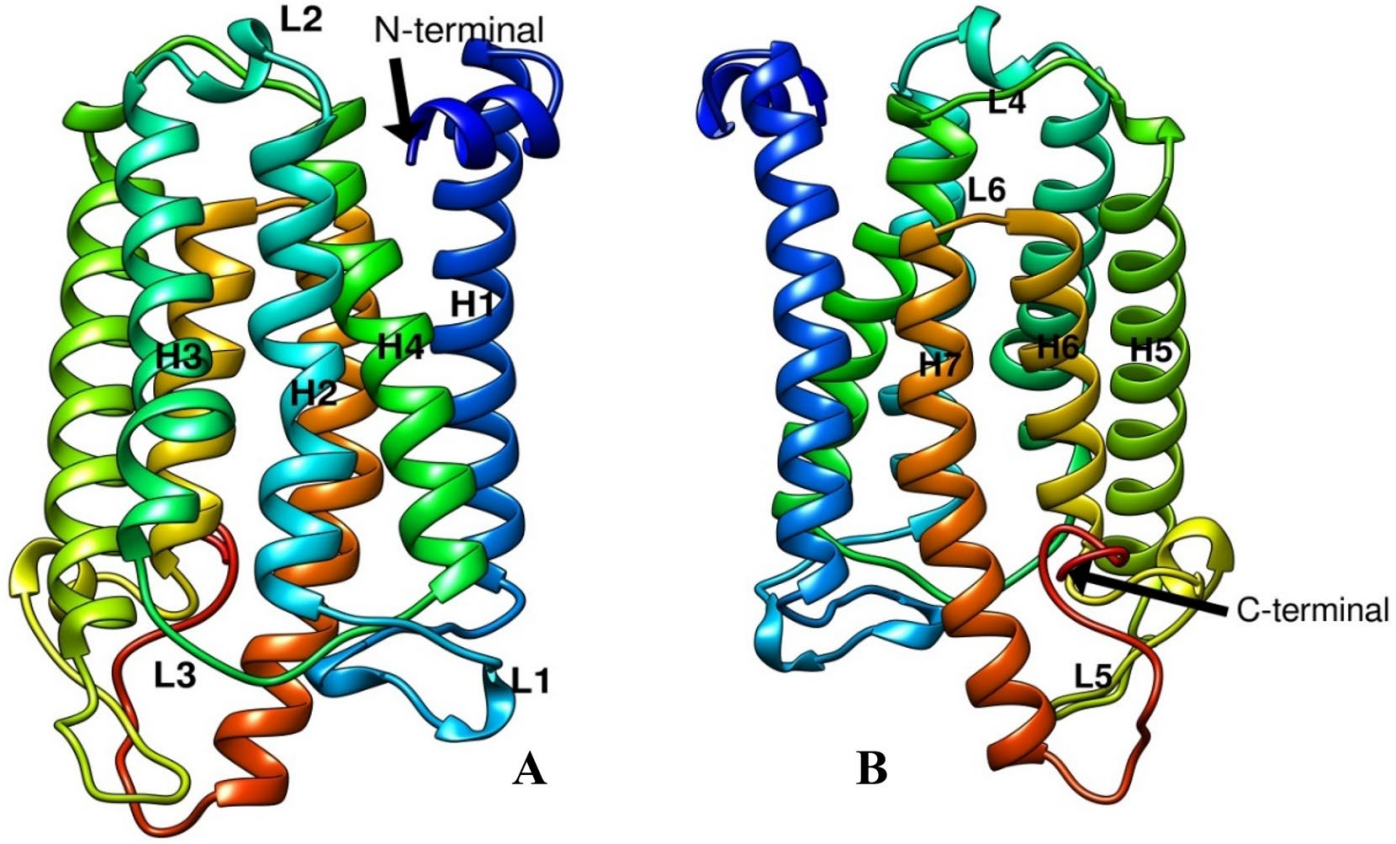
Ribbon diagram showing the tertiary structure of CmSRD5A1 protein in which alpha helices and loops are labelled as H1-H7 and L1-6 respectively and are shown in different colours. A. Shows one face of the structure with N-terminal and H1-H4 and L1-L3. B. Structure A was rotated 180^o^ to show remaining helices H5-H7 and loops L4-L6.

**Figure 8:**
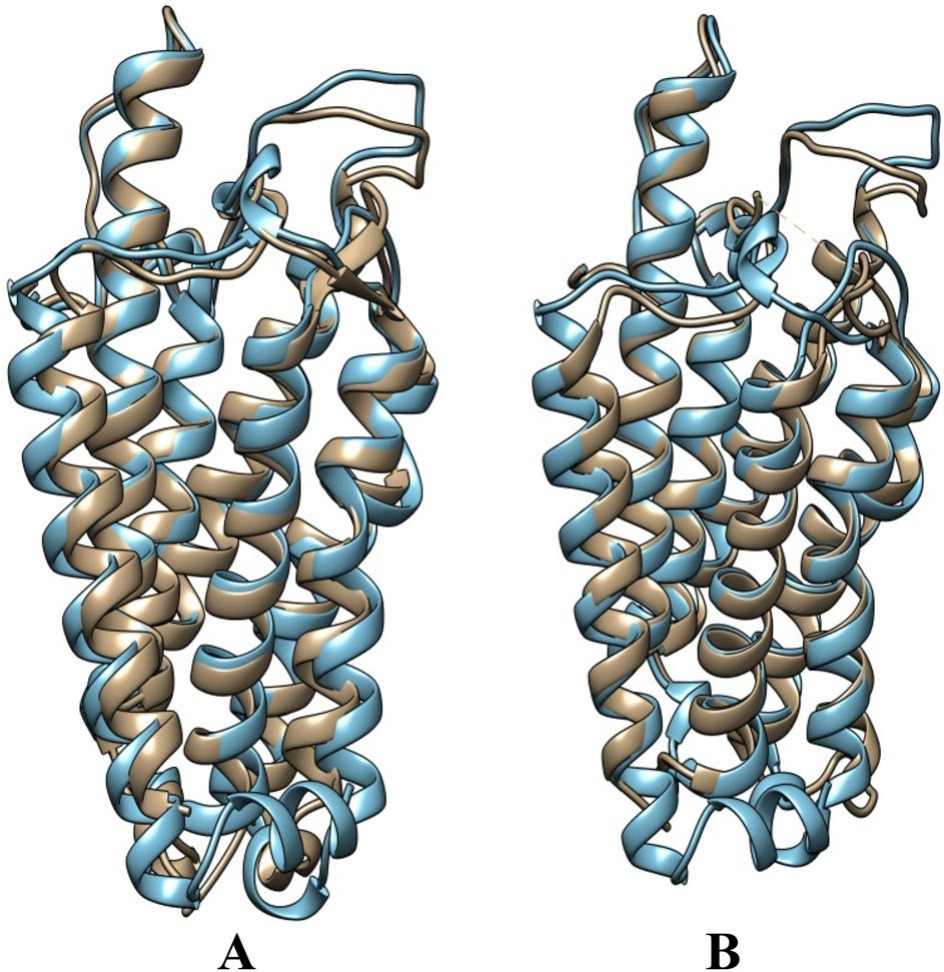
Structural superimposition of CmSRD5A1 with bacterial and human orthologs. (**A**) CmSRD5A1 is superimposed to SRD5A of *Proteobacteria bacterium* (PDB ID. 7c83). (**B**) CmSRD5A1 is superimposed to human SRD5A2 (PDB ID. 7bw1) to understand structural similarity. CmSRD5A1 is coloured in cyan, and the known structures are coloured in gold.

The 3D structure of the CmSRD5A1 sequence generated above was predicted using highly accurate AlphaFold 2 in Google Colab online platform. There were five structures generated. The rank_1 among all five was selected for further analysis. CmSRD5A1 contains seven transmembrane segments (TMs), with the N-termini located on the periplasmic side of the plasma membrane and the C-termini on the cytosolic side (Fig. 7A,B). The 7-TM helices are connected by six loops (L1–6). The surface representation of CmSRD5A1 revealed a large cavity inside the 7-TM domain at the cytosolic side formed by all 7-TMs and L1, L3, and L5. The predicted structure was superimposed to SRD5A of Proteobacteria bacterium (PDB ID. 7c83; Fig. 8A) and human SRD5A2 (PDB ID. 7bw1; Fig. 8B) to understand structural similarity. The observed RMSD for the human structure was 1.363 Å for 245 aligned residues and for the bacterial structure was 1.506 Å for 251 aligned residues. This shows a high degree of structural conservation of fish enzymes.

**Figure 9:**
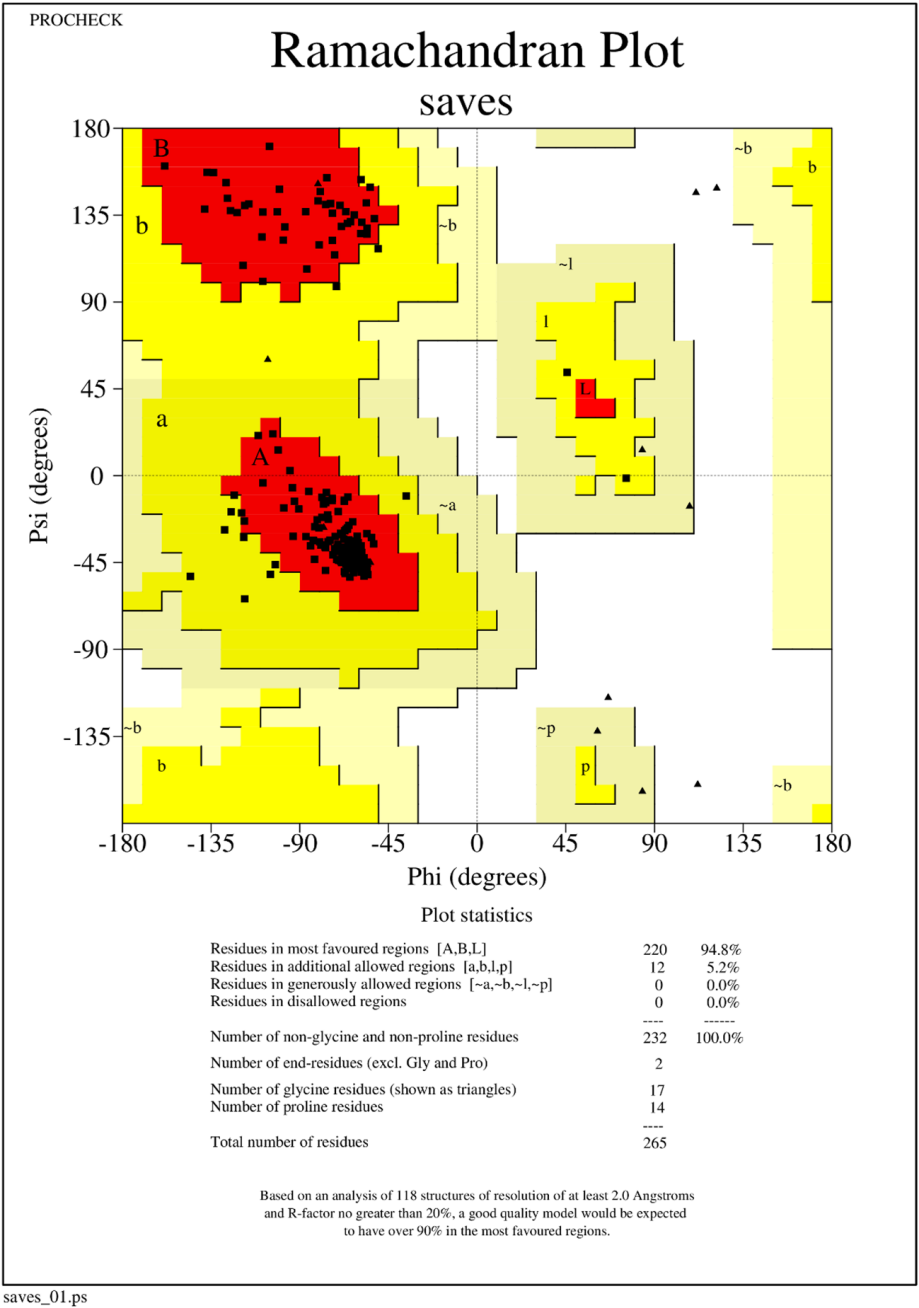
Ramachandran plot CmSRD5A1 showing the dihedral angles Psi and Phi of amino acid residues. The residues which lie in the most favoured regions are shown as black dots in red area and the residues which lie in additional allowed regions are in red dots.

**Figure 10:**
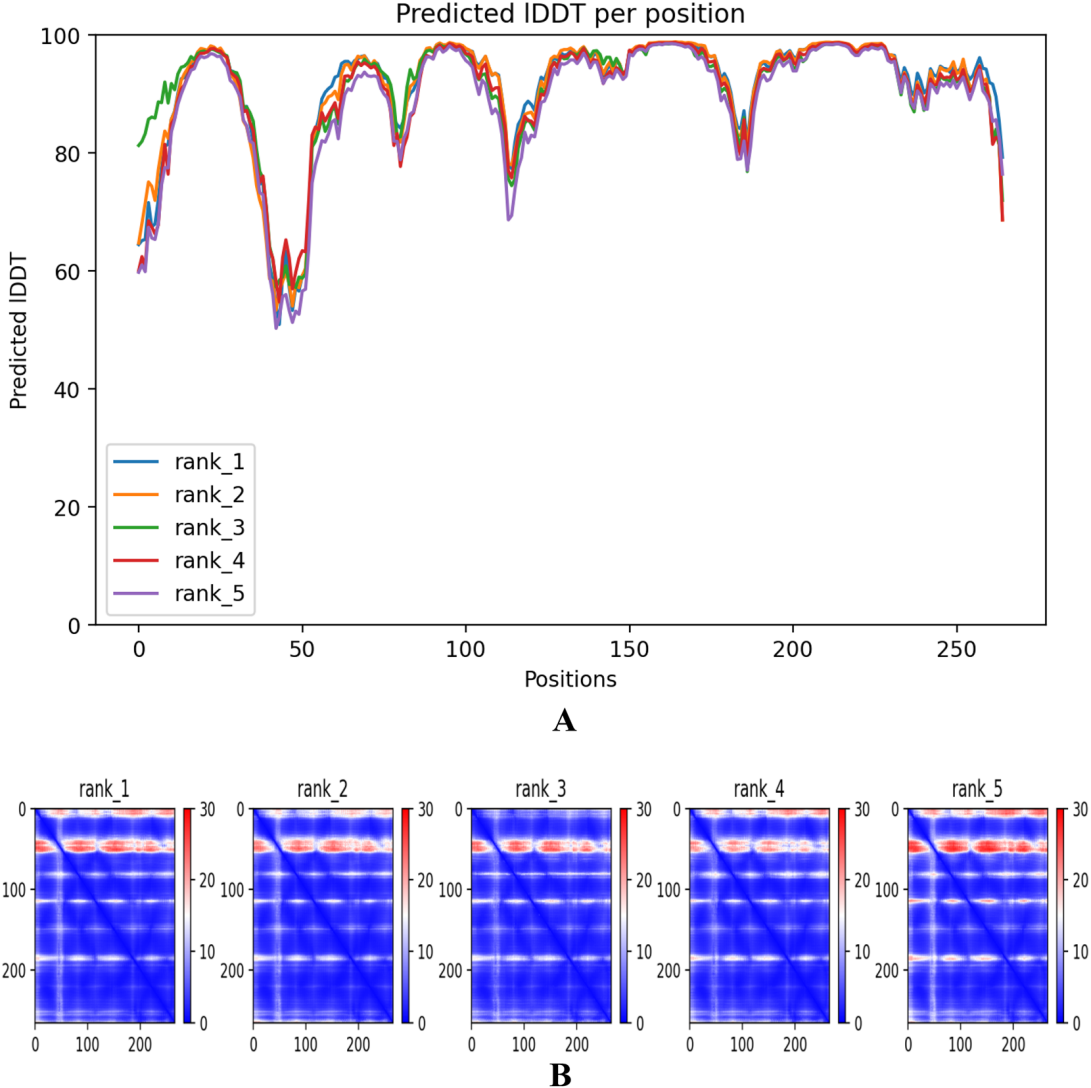
Structural quality parameters given by AlphaFold 2. (**A**) Predicted local distance difference test (pLDDT) plot for five generated structures. (**B**) Predicted aligned error (PAE) plot for predicted CmSRD5A1 structures. Rank_1 structure was used for further analysis.

The quality of the predicted structure was analysed by examining using the Ramachandran plot, pLDDT and PAE scores. Ramachandran plot analysis revealed that 94.8% of the total residues are in the most favoured region and none of them in the disallowed region (Fig. 9). IDDT and PAE plots (Fig. 10A,B) generated by AlphaFold revealed a very good quality structure The N-terminal region around the residues 40–50 was of slightly lower quality with IDDT score of 60. Other regions are of high quality as can be seen in the IDDT and PAE plots.

**Figure 11:**
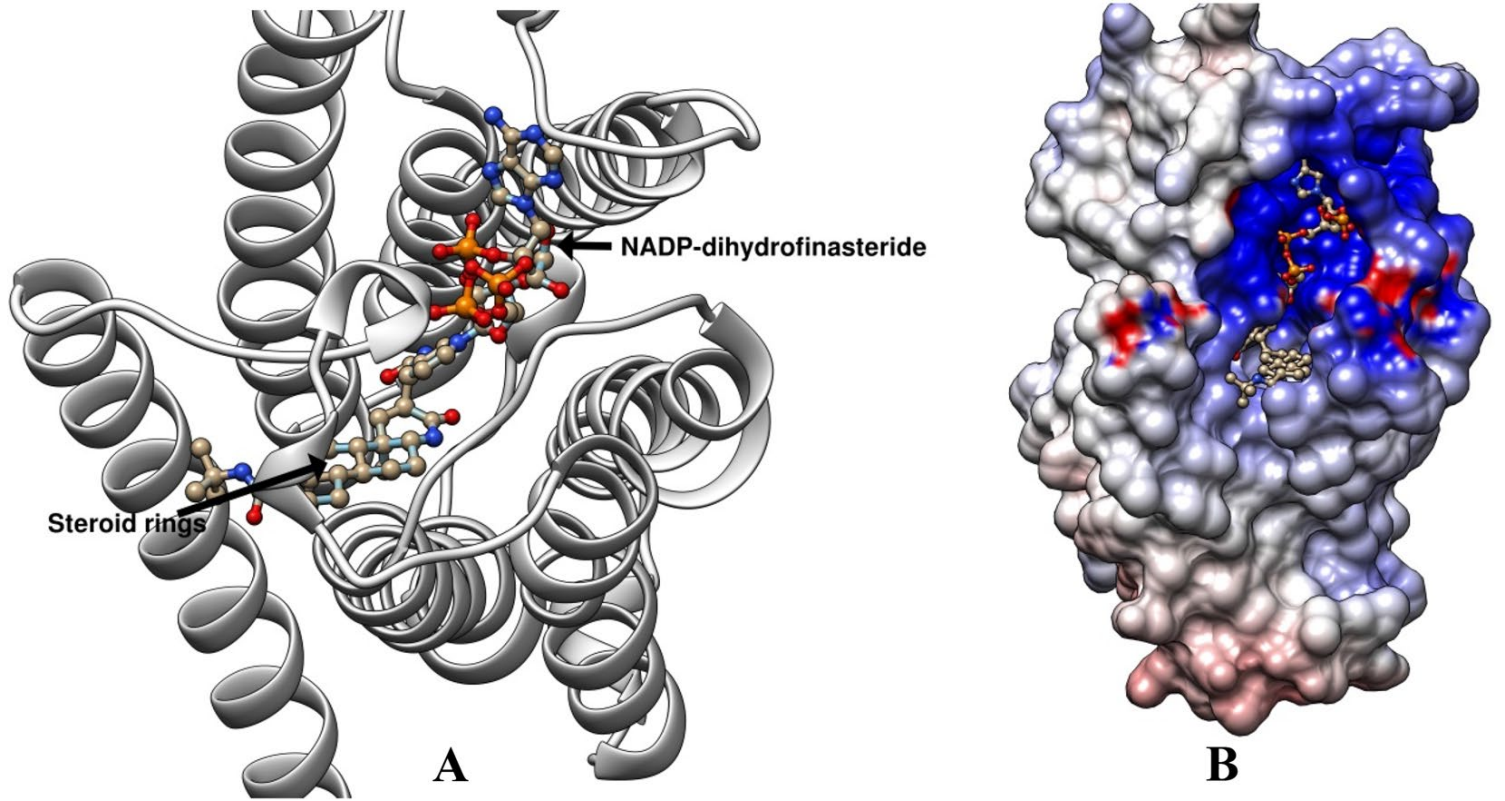
CmSRD5A1 structure having inserted with NADP-dihydrofinasteride based on the structural superimposition with human SRD5A2 (PDB ID: 7bw1). The steroid rings group is also marked.

In the predicted structure it was observed that there is a cavity towards the cytoplasmic side of the enzyme. The cavity has two openings one towards the cytosolic side and the other towards the transmembrane side between the TM1 and TM4. The substrate-binding cavity shows two relatively separate tunnel-like pockets for two ligands. It is reported that this enzyme binds to NADPH and steroid hormones. The ligands NADPH and NADP-dihydrofinasteride (Fig. 11A) were transferred after superimposition from bacterial and human enzymes to understand the ligand interaction. It was observed that NADPH insert into the binding pocket inside the 7-TM bundle. The binding pocket for NADPH is enclosed on the cytosolic side by the cytosolic loops, almost completely shielding it from the cytosol. The nicotinamide-ribose moiety is buried inside 7-TM away from the opening. The interaction of steroid moiety was also revealed by comparing it with the human structure having the complex NADP-dihydrofinasteride (Fig. 11A) The binding mode of steroid is in such a way that its Δ4 double-bond comes in close proximity to the nicotinamide ring. The electrostatic potential analysis revealed that the cytosolic side of the tunnel is highly electropositive but the transmembrane side opening is neutral (Fig. 11B). NADPH is enclosed towards the cytosolic side in highly electropositive side of the tunnel. The steroid hormone binds towards the transmembrane opening in a relatively hydrophobic environment.

### Ligand interaction and catalytic mechanism

**Figure 12:**
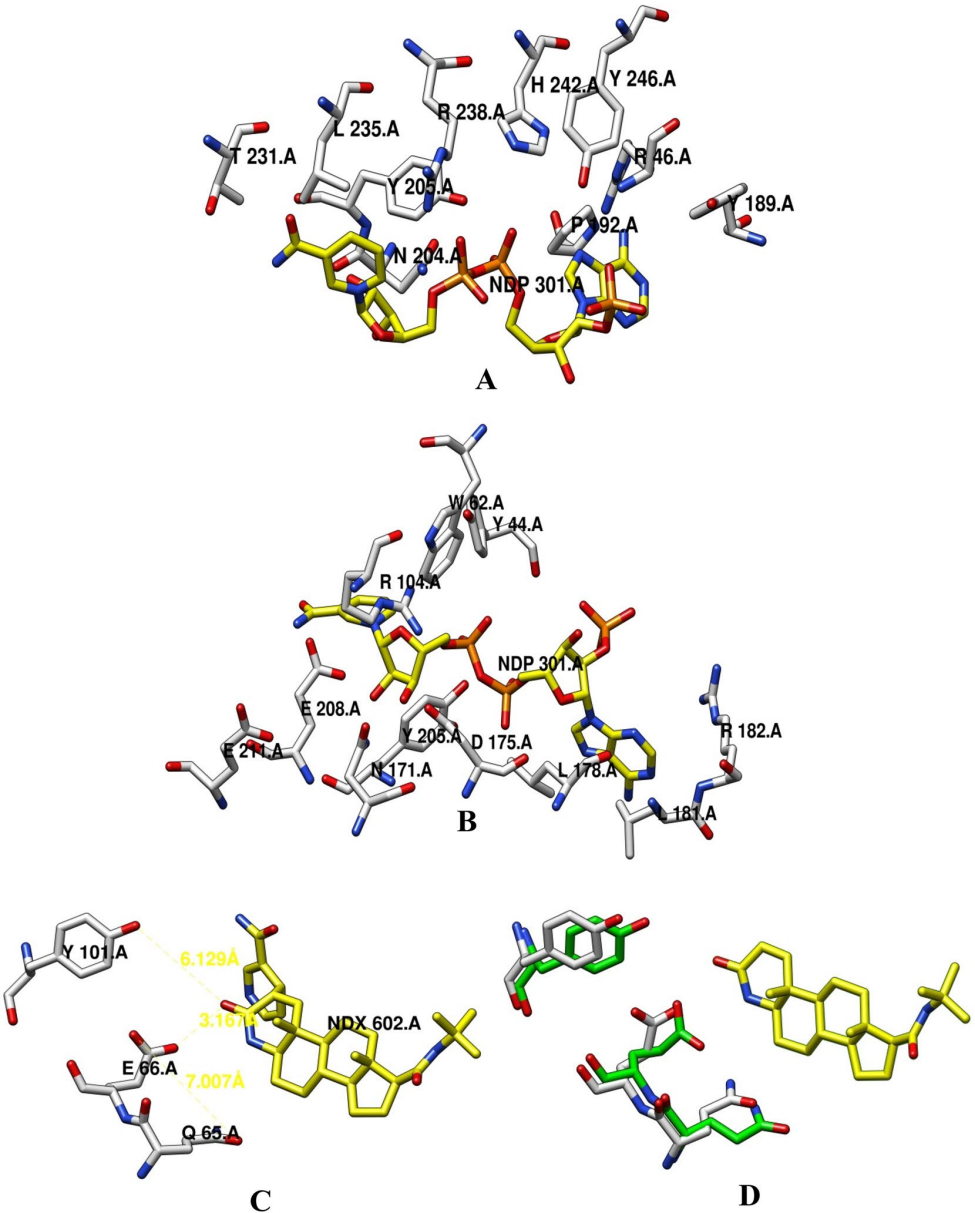
Ligand interaction and catalytic residues based on structural superimposition. (**A**,**B**) Interaction of NADP with amino acid residues predicted based on superimposition and the structural based sequence alignment with *Proteobacteria bacterium* SRD5A (PDB ID. 7c83). (**C**) Catalytic residues involved in H-bonding with ketone group at C-3 of steroid. (**D**) Catalytic residues superimposed with human SRD5A2 ((PDB ID. 7bw1).

Based on the structure based sequence alignments the amino acid residues involved in ligand interaction were revealed. The conserved residues forming hydrogen bonds with NADPH were identified as N171, D175, and R182 on TM5, N204, Y205, and E211 on TM6, and T231, R238, and H242 on TM7 (Fig. 12A,B). The residues on intracellular loop 1 (Y44 and R46) and loop 3 (Y189) are also conserved and can be contributed to NADPH binding through direct hydrogen bonds. Conserve residues partly interacted with NADPH through water mediated hydrogen bonds are R46 on intracellular loop 1, N204 on TM6 and R238 on TM7. Polar residues binding to the ketone group in the steroids were identified as E66 and Y101 (Fig. 12C,D). The hydrophobic residues binding to steroids are not so conserved. The region of the substrate binding pocket involved in binding with steroids contains a conserved signature motif identified as E66 and Y101 important for catalysis. This motif forms a triangular hydrogen bond that coordinates the ketone group at C-3 in the steroids. This coordination brings the steroid into proximity to the nicotinamide of NADPH, leading to the hydride (H −) transfer and Δ4 double-bond reduction. Additionally, Q65 was also identified which can bind and stabilize E66 as reported in human and bacterial enzymes.

### Expression analysis of CmSRD5A1 gene

**Figure 13:**
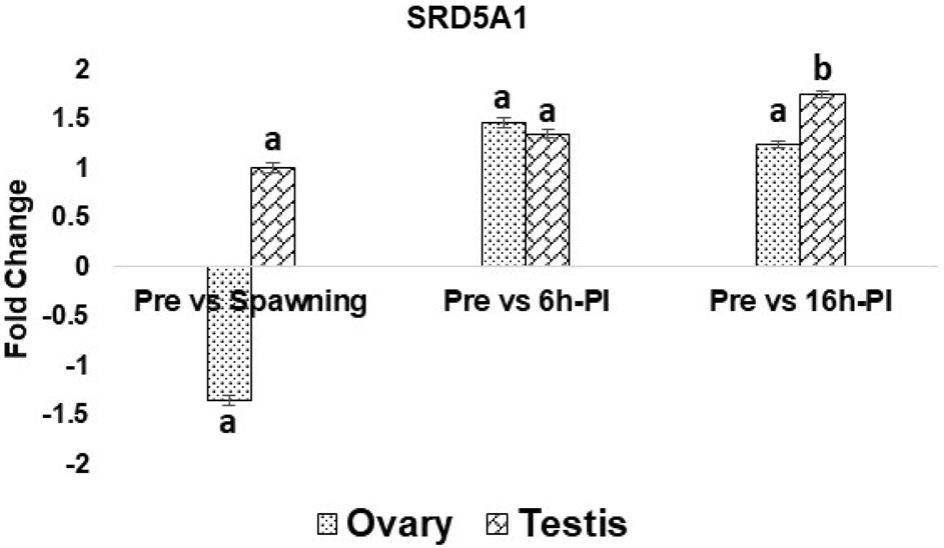
qRT-PCR analysis of SRD5A1 mRNA expression in ovary and testis at different maturation stages of *C. magur*. The relative expression of SRD5A1 normalized with β-actin was calculated using the comparative Ct method. Data for real-time PCR were expressed as mean ± S.E.M (n = 3). The superscripts (a,b,c,d) above the bar represents the statistical significance at P < 0.05.

**Figure 14:**
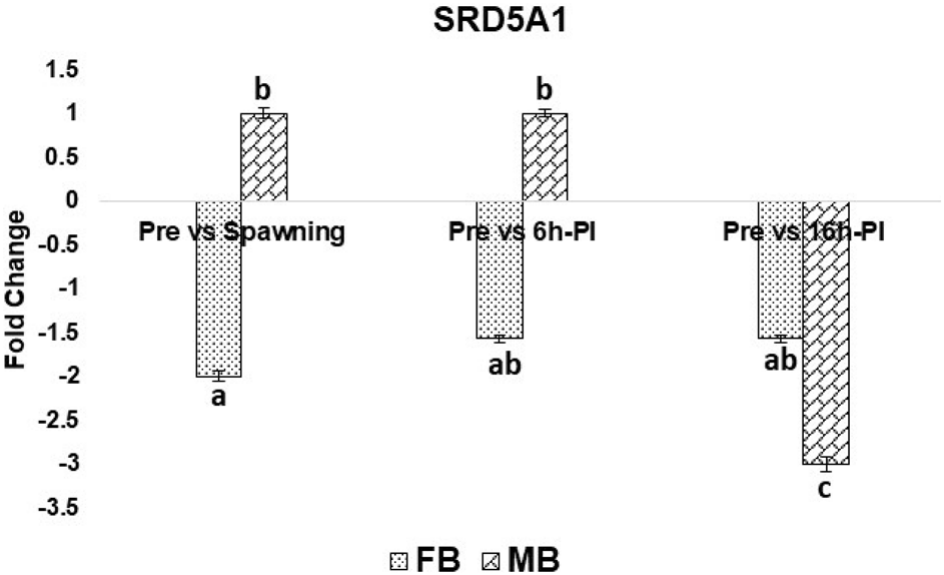
qRT-PCR analysis of SRD5A1 mRNA expression in brain of both the sexes of *C. magur* at different maturation stages. The relative expression of SRD5A1 normalized with β-actin was calculated using the comparative Ct method. Data for real-time PCR were expressed as mean ± S.E.M (n = 3). The superscripts (a,b,c,d) above the bar represents the statistical significance at P < 0.05.

The mRNA expression pattern of SRD5A1 gene in *C. magur* adult male and female fishes was determined through qRT-PCR in the brain and gonads at four different maturation stages namely preparatory, spawning (before injection), 6 h post-Ovatide injection and 16 h post-Ovatide injection (post-spawning). In female fish, the CmSRD5A1 gene was expressed in both the tissues of all stages but significantly higher in the ovary compared to the brain. The expression of CmSRD5A1 in the ovary was decreased at spawning stage compared to the preparatory stage. The highest mRNA expression of CmSRD5A1 in the ovary was found after the 6 h of Ovatide induction (Fig. 13). In testis, significant peak mRNA expression of CmSRD5A1 was observed at 16 h post-Ovatide injection stage compared to the preparatory stage (Fig. 10). Whereas in case of the brain the lowest mRNA expression of CmSRD5A1 in female was obtained at spawning stage which was at peak in preparatory stage (Fig. 11). In the brain of male fish, the mRNA expression of CmSRD5A1 was suddenly dropped at 16 h-PI stage with significantly threefold change (Fig. 14).

## Discussion

The full-length cDNA sequence of the SRD5A1 gene (795 bp ORF) was cloned from *C. magur* using the 5'/3' RACE-PCR strategy. Previous studies have demonstrated the presence of 5α-reductase activity in select fish species such as goldfish, toadfish, lamprey, rainbow trout, and zebrafish^16,40^. Comparative analysis of the CmSRD5A1 amino acid sequence with sequences from other fish and mammal species revealed significant regions of similarity, including a putative NADPH-binding domain. Within this domain, a highly conserved region consisting of 31 amino acids (residues 170–200) was tentatively identified (Fig. 3). Further sequence analysis indicated that CmSRD5A1 contains two conserved histidine residues (H242, H243) in its C-terminal region, which are known to be essential for substrate binding and catalytic activity^41^ (Fig. 3). In a previous report by^42^, a tetrapeptide segment involved in substrate binding was identified in the chimeric SRD5A1 enzyme constructed from human and rat. In our study, a similar tetrapeptide segment (-A-A-C-V-) for steroid binding was also found in CmSRD5A1 (Fig. 3). However, the sensitivity of this segment to inhibitors of steroid 5α-reductase remains unknown. These findings are consistent with previous reports, confirming the high conservation of these motifs^11,43^.

The phylogenetic analysis of CmSRD5A1, with other species, revealed high similarity with *I. punctatus* followed by other fishes like *L. carcerifer, P. nattereri* and *D. rerio*. The *C. magur* SRD5A1 showed 51% similarity with its ortholog from the human.

The hydropathicity analyzed using the Kyte-Doolittle algorithm predicted that CmSRD5A1 protein is more hydrophobic at N-terminus region due to the presence of a highly hydrophobic region of nonpolar amino acids and is believed to function as a transmembrane signal anchor. Similar kind of hydropathy profile has been observed in its ortholog proteins^44^ supporting that CmSRD5A1 protein is hydrophobic. Since the SRD5A1 is a membrane-associated protein with a hydrophobic transmembrane domain, no signal peptide could be identified.

SRD5As belong to a large group of eukaryotic membrane embedded steroid reductases catalyzing the irreversible reduction of the Δ4,5 bond in Δ4-3- ketosteroids using reduced NADPH as the hydride donor cofactor^45,46^. The membrane-embedded SRD5A family in humans includes five members, SRD5A1–3 and glycoprotein synaptic 2 (GSPN2) and GSPN2^46^. There is another group of steroid reductase from the Aldo–Keto Reductase (AKR) superfamily which is soluble and involved in numerous metabolic transformations^47^. There are numerous structures available for soluble steroid reductases but the 3D structure and mechanism of action of eukaryotic membrane-embedded steroid reductases are less explored. To date, there are two structures of SRD5A enzymes namely PbSRD5A from Proteobacteria bacterium^45^ and human SRD5A2^46^ are reported. Although these steroid/sterol reductases share very little sequence similarity, they all use NADPH as the cofactor to reduce specific carbon–carbon double bonds in their steroid substrates. It is not surprising that SRD5As exhibit similar fold and the NADPH cofactor-binding sites are highly conserved in the steroid reductase families. Biochemical and structural studies carried out by^45,46^ on human and bacterial enzymes have led to the understanding of the structural and catalytic mechanism of this enzyme.

Both the reported structures have very high degree of structural similarity. They adopt a monomeric 7-TM structural topology with a largely enclosed binding cavity inside the transmembrane domain. Structure predicted in the current study from the catfish *Clarias magur* which is another vertebrate-like mammal has also shown the same structural topology. Structural superimposition of current structure with above mentioned human and bacterial SRD5A given an RMSD value < 2.0 Å for more than 80% of the residues aligned showing the very high degree of structural conservation. Analysis of surface features revealed a tunnel towards the cytoplasmic side having two openings one towards the lipid bilayer and the other towards the cytoplasm region. The cytoplasmic loop partially covers the cytoplasmic opening. The electrostatic potential calculation revealed that the cytoplasmic region of the cavity is highly electropositive but the membrane side opening is hydrophobic. This type of asymmetry is noted in both the bacterial and human SRD5A structures. The electropositive side of the tunnel is involved in the binding of NADPH and the hydrophobic part binds with steroids. The ligands were transferred to CmSRD5A1 from known SRD5A structures.

Ligand binding and catalytic residues were identified based on structural superimposition based sequence alignment in the predicted CmSRD5A1. The amino acid comparison with bacterial enzyme revealed almost conserved residues for NADPH binding. On the other hand, residues involved in steroid binding are not well conserved, but the locality was hydrophobic. Structural, computational and mutagenesis studies reveal the molecular mechanisms of catalysis and finasteride inhibition involving residues E57 and Y91 in human SRD5A1^46^. The corresponding catalytic identified as E66 and Y101 are important for catalysis. This motif forms a network of hydrogen bonds that coordinate the ketone group at C-3 in the steroids. This coordination brings the steroid into proximity to the nicotinamide of NADPH, leading to the hydride (H −) transfer and Δ4 double-bond reduction.

Two maturation stages and two after 6 h and 16 h Ovatide injected stages of *C. magur* adult male and female fishes were taken for CmSRD5A1 mRNA expression study by qRT-PCR. The differential mRNA expression obtained for the ovary was not statistically significant. In the ovary, the CmSRD5A1 gene showed lower mRNA expression at the spawning stage compared to the preparatory stage while expression peaked at 6 h post-Ovatide injection stage. This mRNA expression profile in the ovary suggested that more CmSRD5A1 at preparatory stage leads to the conversion of more testosterone into DHT. It has been reported that DHT stimulates the secondary sexual characteristics and masculinization of the female genotype to a higher than testosterone^48^. However, the actual physiological role of testosterone and DHT have not yet been fully described in female fish but considered that the higher production of CmSRD5A1 might alter the reproductive hormonal secretion in the female through the accumulation of DHT. On the other hand, DHT can increase the 17β-estradiol output from the teleost ovary^40^ which is the principal estrogen secreted in all vertebrates including fish and is essential for the development and maintenance of female reproductive tissues like the ovary^49,50^. Therefore the lower expression of CmSRD5A1 at the mature stage in the ovary as well as in the brain of female fish resulted in less accumulation of DHT which might reduce the production of 17β-estradiol. In agreement with the earlier studies^51,52^ the mRNA expression of CmSRD5A1 in the male brain was observed and found higher than in the testis. In the case of the male brain, the mRNA expression of CmSRD5A1 was found to be down-regulated significantly (P < 0.05) after 16 h of Ovatide injection. The lower expression of CmSRD5A1 in the male brain at 16 h post-Ovatide injection stage resulted in less availability of DHT which might be the possible reason for the dysfunction of the testis in captivity. As reviewed^15^, different several androgen receptors have been characterized in fishes, but none is specific for KT indicating that some other androgens like DHT might be involved in the mediation of the androgenic response in different target tissues. Few studies have reported an androgen receptor with high binding affinity to DHT in many teleost species and suggested DHT is often one of the most potent inducers of androgen receptor transcriptional activity in vitro^53,54^. If DHT is the more potent androgen and preferred ligand for androgen receptor (AR) transactivation than T and KT, the overexpression of DHT through the SRD5A1 activation might help in spermatogenesis.

## Conclusion

In summary, the present study reports the full-length coding sequence of the SRD5A1 gene from *C. magur*. The deduced amino acid sequence was analysed using various bioinformatics tools to predict the structural and functional features. The conserved catalytic residues involved in Δ4 double-bond reduction are identified as E66 and Y101. The differential mRNA expression analysis of CmSRD5A1 revealed the highest expression in the ovary at 6 h post-Ovatide injection stage and in testis at 16 h post-Ovatide injection stage compared to the preparatory stage. In both sex gonads, the lowest expression of CmSRD5A1 was observed at the spawning stage. Whereas in the case of the brain, the lowest mRNA expression of CmSRD5A1 was obtained at spawning stage in females and males at 16 h post-Ovatide injection stage. As per our knowledge, this is the first report of cloning and structural analysis of CmSRD5A1 full-length cDNA in Indian catfish. In general, this study is a basis for further exploration of structural and functional characteristics of the SRD5A1 gene in *C. magur*, including its protein structure and involvement in signal transduction. The expression pattern of the transcript in gonads and during different reproductive phases provides insights into their involvement in reproductive cycles. Its actual role during the reproductive phases needs to be explored in further research.

## Data Availability

Data will be made on special request to the corresponding author. However, the complete CDS sequence derived from this study has been submitted to NCBI GenBank (Accession number: LC383722 {Protein Id: BBE00816}) which is available there.
